# The Role of T Cells in Hematopoietic Stem Cell Engraftment

**DOI:** 10.1100/tsw.2006.47

**Published:** 2006-02-26

**Authors:** Elizabeth Hexner

**Affiliations:** Division of Hematology/Oncology, University of Pennsylvania, Philadelphia, PA 19104, USA

**Keywords:** stem cell transplantation, engraftment, umbilical cord blood, T cells

## Abstract

Much attention has focused on the immune recovery of donor T cells following hematopoietic stem cell transplantation (HSCT). Termed immune reconstitution, a better understanding of the dynamics of the functional recovery of immune cells following HSCT has important implications both for fighting infections and, in the allogeneic setting, for providing antitumor activity while controlling graft-vs.-host disease (GVHD). The immune cells involved in immune reconstitution include antigen-presenting cells, B lymphocytes, natural killer cells, and, in particular, T lymphocytes, the immune cell that will be the subject of this review. In addition, T cells can play an important role in the process of engraftment of hematopoietic stem cells. The evidence for a T cell tropic effect on hematopoietic engraftment is both direct and indirect, and comes from the clinic as well as the research lab. Animal models have provided useful clues, but the molecular mechanisms that govern the interaction between donor stem cells, donor T cells, the host immune system, and the stem cell niche remain obscure. This review will describe the current published clinical and basic evidence related to T cells and stem cell engraftment, and will identify future directions for translational research in this area.

## HOST AND DONOR T CELLS ARE PIVOTAL FOR ENGRAFTMENT: EARLY EVIDENCE

Without conditioning of a recipient using chemotherapy, radiation, or some combination of these two modalities, a donor graft is rejected. Antigenic differences between donor and host, in addition to T cells, are key variables determining the success of engraftment. In mice, T cell depletion of bone marrow grafts decreases functional stem cell numbers approximately 10 fold in major histocompatibility(MHC)-matched, minor histocompatibility-mismatched recipients and as much as 100-fold for MHC-mismatched hosts. [1]. Increasing doses of total body irradiation can overcome rejection of T cell depleted (TCD) grafts in mice, even across MHC barriers[1,2,3]. Likewise in human studies, higher doses of total body irradiation are inversely correlated with graft failure, and histocompatibility differences have the effect of reducing the number of functional stem cells, with a graft failure rate of 10–30% in recipients of MHC-identical TCD grafts and 50–70% in recipients of MHC-mismatched TCD grafts[4]. One current approach to the depletion of functional stem cells in the TCD setting is to use high doses of stem cells when possible. There is evidence that a subset within the CD34+ stem cell–enriched population of cells, termed “veto cells” has tolerance-inducing properties and may be of potential use in settings such as haploidentical hematopoietic stem cell transplantation (HSCT), where antigenic differences between donor and host are considerable[5].

The importance of host immune barriers was also defined by the HSCT experience in patients with severe aplastic anemia (SAA), an acquired bone marrow failure syndrome that appears, at least in some cases, to be characterized by immune dysregulation. Somewhat surprisingly, a retrospective registry analysis looked at HSCT in SAA in identical-twin donor recipient pairs (syngeneic HSCT) — a unique circumstance where one would expect immune barriers to be absent — and found that the majority of transplanted grafts failed when transplanted without immunosuppressive conditioning of the recipient[6]. In the more common allogeneic scenario, (where the donor, often related, is matched for MHC determinants but is otherwise not identical) the Seattle group determined risk factors for graft rejection in SAA. These included low cell dose, prior transfusions, positive *in vitro* testing for an alloimmune response, and year of transplantation — most likely a reflection of the change in transfusion practices in SAA patients[7]. Taken one step further, and with supporting evidence from mice and dogs, these investigators found that if donor bone marrow cells were cotransplanted with donor white cells, rates of engraftment improved and overall survival was prolonged, despite the increased incidence of graft-vs.-host disease (GVHD)[8,9]. In 1982, the authors speculated that possible mechanisms for graft facilitation by donor white cells included an increase in absolute numbers of donor stem cells, a graft-vs.-host reaction, or a unique interaction between lymphocytes and the donor bone marrow; while stem cells and lymphocytes are more precisely defined and our terminology more refined today, the putative mechanisms remain largely unchanged.

The immune dysregulation in SAA may augment host barriers, as the incidence of graft rejection is considerably higher for SAA than for other indications for alloHSCT, where graft rejection is a rare complication. Nevertheless, taken together with other experimental evidence, the SAA experiences illustrate not only that overcoming host rejection by diminishing host immunity (i.e., with irradiation or chemotherapy) is required for the engraftment of donor cells, but that donor white cells, including T cells, are also critical to the process. Whether their role is simply to overcome residual host immune barriers through cytotoxic mechanisms or to interact with the stem cell niche to facilitate stem cell engraftment through other mechanisms is not known.

## T CELLS IN CLINICAL STEM CELL TRANSPLANTATION: THE YIN AND THE YANG

Because T cells mediate GVHD, a major toxicity of allogeneic stem cell transplantation (alloHSCT), depleting stem cell grafts of T cells was initially an attractive approach to prevent GVHD. Indeed, early analyses of alloHSCT from unrelated donors showed that T cell depletion decreased rates of acute GVHD and, in at least one retrospective study, was actually associated with improved early myeloid engraftment, but ultimately with an unexpectedly high rate of secondary graft failure[10]. In fact, the positive association between TCD and early engraftment in this retrospective registry analysis is more likely a reflection of differences in other transplant-related factors (i.e., myelosuppressive immunosuppressive medications given to recipients of non-TCD grafts) rather than a true biologic effect. In aggregate, studies of TCD alloHSCT have shown that TCD alloHSCT is actually associated with a significant delay in engraftment and high rates of graft failure, with some studies reporting failure rates exceeding 50%, as compared to less than 1% in unmanipulated stem cell grafts. It should be noted that techniques of depletion of T cells vary, both in the effectiveness of depletion of T cells and in the removal of functional stem cells in the process[11,12,13,14]. Finally, in addition to graft failure, the development of post-transplant lymphoproliferative disorders and, most importantly, relapse of disease are more common in TCD alloHSCT; these adverse effects combine to deprive TCD of any clear overall survival benefit when compared to conventional approaches (reviewed in [4]).

Collectively, these experiences have prompted investigators to attempt to define more precisely the graft-facilitating effect of donor T cells, raising the following questions:

- What can the cellular makeup and engraftment kinetics of differing sources of hematopoietic stem cells teach us about the role of T cells in engraftment?
- Are there subsets of donor T cells with greater graft-facilitating potency? If so, by what mechanisms do these cells exert this effect?
- What are the relative contributions of recipient vs. host T cells in stem cell engraftment?

## What Can the Cellular Makeup and Engraftment Kinetics of Differing Sources of Hematopoietic Stem Cells Teach Us About the Role of T Cells in Engraftment?

Not all sources of stem cells behave identically, as exemplified by the clinical experience with umbilical cord blood (UCB) HSCT. UCB is abundant, safe to obtain, and highly enriched in stem cells. Recipients of UCB tolerate a greater degree of HLA mismatching without an increased incidence of GVHD and, unlike alloHSCT using peripheral blood or bone marrow stem cells, outcomes with UCB transplantation are not directly correlated with histocompatibility differences[15]. Despite these advantages, UCB HSCT, at least in Western countries, is reserved as an alternative stem cell source for adults for whom a matched related or unrelated donor is unavailable, because outcomes thus far are inferior, despite lower rates of acute GVHD and comparable rates of chronic GVHD[15,16]. The major limitation of UCB transplantation in adults has been a relatively high incidence of both primary graft failure and an overall delay in engraftment. There is no doubt that limiting cell numbers in stored UCB grafts are the major contributor to graft failure in adult recipients[17]. The delay in engraftment may also be due to limiting cell numbers, hence the intensive investigation focused on *ex vivo* stem cell expansion (reviewed in [18]). Alternatively, or in addition, UCB stem cells may grow more slowly or have higher cytokine requirements than do peripheral blood or bone marrow stem cells, although *in vitro* and *in vivo* preclinical data suggest just the opposite[19,20,21]. Another possibility is that UCB T cells or other accessory cells in the graft account for the delay. UCB T cells are distinct from adult T cells: unlike peripheral blood or bone marrow T cells, T cells in UCB are predominantly naïve, i.e., they have not encountered the antigen for which their T cell receptor is specific, an event that triggers a cascade of differentiation and activation events in a T cell. In addition, functional studies have shown that UCB T cells proliferate when alloantigens are encountered, but they demonstrate an impaired secondary proliferative response[20,22]. While these facts may be “true, true, and unrelated”, recent clinical data strengthen the link between UCB stem cells and T cells. In an effort to increase the number of cells in the UCB graft, 23 subjects (median age 24) with hematologic malignancies underwent HSCT using two partially HLA-matched UCB units[23]. In all cases, one UCB unit ultimately predominated. Neither the number of CD34+ cells in the graft nor total cell number predicted which graft would predominate. There was, however, a direct association between the number of T cells and the predominating UCB unit. Interestingly, there were no graft failures in this series of patients, and while this was a phase I/II study, the median time to engraftment (23 days) was meaningfully shorter than that in most published reports of UCB HSCT. Although the reason for this preliminary success is not certain, one interesting possibility is that an alloresponse between UCB units facilitates engraftment, either directly through specific T cell interactions or via nonspecific cytokine-mediated mechanisms. It is notable that in preclinical mouse models, the predominance of one UCB graft over another can be attenuated by depleting both grafts of T cells and other lineage-committed cell types, providing further evidence that accessory cells rather than the stem cells themselves play a key role in determining the pace and ultimate success of engraftment[24]. Nevertheless, the direct link between T cells and UCB engraftment has yet to be identified.

The Japanese experience with UCB HSCT is distinct from the Western experience and is instructive because it suggests that antigenic diversity remains an important determinant of successful UCB HSCT. Like the preclinical mouse data demonstrating that functional stem cell number is influenced by both histocompatibility barriers and the presence of T cells, the more limited diversity of major and minor histocompatibility antigens in Japanese populations may translate into a more enriched functional stem cell compartment[25,26]. In the Tokyo experience, while engraftment is also delayed when transplantation of UCB is compared to that of stem cells from matched unrelated donors (MUD HSCT) in adults, the delay is significantly shorter: the median time to platelet engraftment is 40 days and neutrophil engraftment 22 days, as compared to results analyzed from the International Bone Marrow Transplant Registry, for which median engraftment of these lineages in adult recipients is 60 and 27 days, respectively. Perhaps more importantly, outcomes in terms of disease-free survival and transplant-related mortality are significantly lower in UCB HSCT compared to MUD HSCT in the Tokyo experience, whereas the opposite is true in the published experience in Western countries[15,16,26]. Since limiting cell numbers are a major limitation of UCB HSCT, these differences may simply reflect the smaller average weight of the Tokyo patient population[26]. In any case, all these data should be interpreted with some caution; coming either from single-institution experiences or retrospective registry analyses, the possibility exists that differences in patient selection alone could account for most of the observed differences. However, common to the published adult UCB literature (Eastern and Western) is one reassuring, and not necessarily expected, outcome when knowing that UCB T cells have uniquely tolerizing properties: disease relapse is not excessive, as it has been for TCD HSCT, supporting the idea that UCB immune cells retain graft-vs.-tumor potency.

Experience with more conventional stem cell sources — mobilized peripheral blood stem cells (PBSC) and bone marrow cells (BM) — has shown that on average, PBSC grafts contain more than tenfold the number of T cells than do BM (and three- to fourfold the number of CD34+ cells), and tend to engraft faster than do BM cells. PBSCs, although naturally present in low numbers, are mobilized from the bone marrow into the peripheral blood of donors. This is typically accomplished by using growth factors such as G-CSF or GM-CSF, which are also known to have modulatory effects on antigen presenting cells, and directly or indirectly on T cells[27,28]. Despite higher T cell numbers, rates of acute GVHD are not increased in PBSC transplantation, although the probability of developing chronic GVHD does appear to be higher (reviewed in [28]). One might speculate that the higher ratio of T cells to stem cells in PBSC confers a stronger graft-promoting effect, or that qualitative differences in cells harvested from the periphery account for the acceleration of engraftment, but to date there is no direct mechanistic link between T cells and engraftment.

## Are There Subsets of Donor T Cells with Greater Graft-Facilitating Potency? If So, by What Mechanisms do These Cells Exert this Effect?

Mouse models of human stem cell transplantation have demonstrated that donor CD8+ T cells prevent graft rejection via perforin-mediated, and, to a lesser extent, Fas ligand–mediated mechanisms[29]. Similarly, studies in dogs have shown that dog leukocyte antigen (DLA)–mismatched BM transplants are reproducibly rejected, but that rejection can be overcome by the infusion of both CD4+ and CD8+ T cells[30]. In humans, CD4+ T cells are thought to be key mediators of antitumor activity, but studies of depletion of CD8+ T cells show not only a decreased risk of GVHD, as might be predicted, but also an increased rate of graft failure when compared to unmanipulated grafts[31].

Studies in animals of more specific T cell subsets selected by various cell surface markers have suggested that naïve, nonantigen-educated T cells indeed exert distinct effects on stem cell transplants, including their relative ability to cause GVHD, mount an antileukemic response, and influence immune reconstitution. In one study in mice, effector memory T cells (defined by the lack of the cell surface molecule CD62L) markedly improved T cell recovery after allogeneic transplantation when compared to naïve T cells (CD 62L+) or unseparated T cells[32]. This recovery was the result of engraftment, not simply peripheral expansion of infused T cells, since the repopulating lymphocytes were shown to be bone marrow derived. Importantly, the memory T cell subset did not cause GVHD, whereas naïve donor T cells did. A similar phenomenon — memory-like T cells that appeared not to cause GVHD and naïve T cells that were potent GVHD inducers — were observed in at least two different murine transplant systems by other investigators[15,33,34]. Taken together, these results suggested that activated, memory T cells may be able to support allogeneic stem cell engraftment without increasing the risk of GVHD.

More recent data suggest even more complexity in the different roles played by CD8+ memory vs. naïve T cells, in causing GVHD: first, memory cells obtained from transplanted mice and transferred to secondary recipients (and, therefore, functionally defined) induced severe GVHD; second, a small subset of cells with what was conventionally believed to be a naïve phenotype have now been identified as a "memory stem cell" pool capable of self-renewal, differentiation, and induction of GVHD in mouse HSCT models. Analogous populations in humans have yet to be identified and their function with respect to engraftment is not yet known[35,36].

Other potential subsets of potent graft-enhancing cells include the previously mentioned "veto cells" that have been described in both the CD34+ and CD8+ compartment; and regulatory T cells[5,37]. For the latter, even regulatory cells of third-party origin (neither host nor donor) appear to enhance engraftment in experimental models and have been proposed as an "off-the-shelf" cellular therapeutic[38]. Finally, a population of cells termed "facilitating cells" (FCs) have been defined by their cell surface expression of CD8 and absence of T cell receptor expression[39,40]. While these are not strictly T cells (and may actually be plasmacytoid precursor dendritic cells, specialized antigen-presenting cells), they may be codepleted or copurified with CD8+ T cells, and may in fact be an as yet overlooked subset of graft-facilitating cells. Further investigation into these cells is likely to provide important insights into the key interactions between immune cells, stem cells, and the stem cell niche.

## What Are the Relative Contributions of Recipient vs. Host T Cells in Stem Cell Engraftment?

The focus of current research has largely been on the role of donor T cells in stem cell engraftment. Recently, the impact of depletion of recipient cells has also been investigated. Using a mouse model of autoimmune diabetes, which can be prevented with alloHSCT, depletion of recipient CD8+ cells by administration of anti-CD8+ antibodies prior to alloHSCT facilitated engraftment of donor cells and reduced the radiation threshold that allowed engraftment, with the suggestion that costimulatory interaction between T cells and antigen-presenting cells through the CD40 - CD40 ligand pathway was involved[41]. By contrast, depletion of recipient CD4+ T cells had the opposite effect, inhibiting engraftment and increasing the required dose of radiation, as did depletion of cells using an antibody against the T cell receptor. In a related study using the same mouse model, it was found that recipients preconditioned with an antibody specific for CD3 without irradiation followed by transplant of donor BM and high doses of CD8+ cells (5–20 million/mouse) could successfully engraft. The diabetic phenotype was corrected without GVHD. These findings may not ultimately be generalizable for therapeutic purposes in recipients without autoimmune diseases, but further studies will provide further clues to the process of tolerance induction, and could make alloHSCT for severe autoimmune diseases more standard therapy. In contrast to these data, a study of an inhibitor of CD4+ T cell activation in a different mouse transplant model system (not an autoimmune model) suggested that inhibition of recipient CD4+ T cell activation enhanced engraftment of donor lymphoid and myeloid cells[42]. The conflicting data may be due to the differences in the model systems and the approach to CD4 inhibition, and one might speculate that the relative roles of CD4 T cell subsets (i.e., regulatory T cells) are either different in the two systems or differentially inhibited. Until the interaction between T cells — donor or recipient, memory, naïve, effector, regulatory — and stem cells is understood at a molecular level, these conflicting data (not unique to the preclinical transplant literature) will not be resolved.

The clinical experience with antibody depletion of recipient cells in human alloHSCT suggests that relatively nonspecific recipient immune cell depletion enhances engraftment of donor cells. Alemtuzumab is a monoclonal antibody against CD52, an antigen present on T cells as well as B cells and other antigen-presenting cells. It has been used both for *ex vivo* T cell depletion of stem cell grafts and has been administered directly to patients as part of conditioning regimens for alloHSCT[43,44,45]. Anti-CD52 therapy has been shown to reduce the risk of acute GVHD, although in one study engraftment was delayed, which may be due to the relatively long half-life of the antibody and thus a donor T

cell–depleting effect. In a more recent study of recipients of nonmyeloablative alloHSCT (see below), engraftment was achieved for all but one patient receiving alemtuzumab[44,45]. Because host antigen-presenting cells are also known to be critical to GVHD induction, alemtuzumab may work by blocking this key activation event[46]. Whether the antibody will adversely affect graft-vs.-tumor activity is not known. In any case, there are now conceptual and empirical underpinnings for depletion of recipient immune cells to modulate engraftment and post-alloHSCT events, although the optimal timing and precise role of this approach has yet to be determined.

## FUTURE DIRECTIONS AND CONCLUSIONS

AlloHSCT is currently the only potential cure for many malignant and inherited diseases, yet the donor pool is limited and the treatment-related risks of the procedure are so high that its application is limited to a narrow group of patients: those with the most severe diseases, those with appropriate donors, and younger patients. Defining more precisely the role of T cells in engraftment, in GVHD and in graft-vs.-tumor activity, will potentially broaden the application of alloHSCT and will be of particular importance in the emerging field of nonmyeloablative alloHSCT. This approach, in which the intensity of pretransplantation conditioning is significantly reduced, relies almost entirely on the immune function of donor cells for the eradication of residual disease. The reduced intensity decreases pretransplantation-conditioning toxicity, rendering transplantation more feasible for older patients and those who are not otherwise candidates for myeloablative HSCT. While this approach shows great promise, GVHD and graft failure remain significant problems. A recent analysis of graft composition prospectively correlated peripheral blood stem cell grafts and the achievement of full-donor chimerism following nonmyeloablative conditioning. Analogous to the dual UCB experience, while there was no relationship between number of CD34+ cells and conversion to full-donor chimerism, higher numbers of T cells in a graft (specifically CD8+ T cells) did correlate with full-donor chimerism[47]. It is also known that the delayed infusion of donor lymphocytes following nonmyeloablative alloHSCT for a failing graft can effectively convert recipients into full-donor chimeras, again highlighting the key relationship between T cells and the engraftment of stem cells[48].

In the 1970s, it was shown that T cells were required for the proliferation of primitive red blood cell precursors *in vitro*, and that a soluble factor from the T cell culture was responsible for this effect[49]. In the current era, the dependence of normal hematopoiesis on CD4+ T cells, and specifically activated CD4+ T cells, has been elegantly demonstrated in a mouse model[50]. If T cells and stem cells are so intimately involved during normal hematopoiesis, it should come as no surprise that T cells can also influence transplanted stem cells. As early as the late 1960s, it was shown in a rat HSCT model that thymocytes enhanced hematopoiesis and, somewhat surprisingly, that this property was present and uniquely potent in tolerized thymocytes[51]. A more precise definition of the relationship between T cells and engraftment, identification of a potent subset of graft-facilitating T cells, and the mechanisms involved should translate into meaningful improvements in clinical outcomes in HSCT and might even contribute to a more complete understanding of normal hematopoiesis.

## ACKNOWLEDGMENT

Thanks to Dr. Steve Emerson for his helpful insights and review of the manuscript.

## References

1. Ferrara, J. et al. (1987) Engraftment following T-cell-depleted marrow transplantation. I. The role of major and minor histocompatibility barriers. *Transplantation* **43(4),** 461–467.
2. Ferrara, J.L. et al. (1987) Engraftment following T-cell-depleted bone marrow transplantation. II. Stability of mixed chimerism in semiallogeneic recipients after total-body irradiation. *Transplantation* **44(4)**, 495–499.

3. Ferrara, J.L. et al. (1988) Engraftment following T cell-depleted bone marrow transplantation. III. Differential effects of increased total-body irradiation on semiallogeneic and allogeneic recipients. *Transplantation* **45(5)**, 948–952.
4. Ho, V.T. and Soiffer, R.J. (2001) The history and future of T-cell depletion as graft-versus-host disease prophylaxis for allogeneic hematopoietic stem cell transplantation. *Blood* **98(12)**, 3192–3204.
5. Reisner, Y. and Martelli, M.F. (2000) Transplantation tolerance induced by "mega dose" CD34+ cell transplants. *Exp. Hematol.* **28(2)**, 119–127.
6. Hinterberger, W. et al. (1997) Results of transplanting bone marrow from genetically identical twins into patients with aplastic anemia. *Ann. Intern. Med.* **126(2)**, 116–122.
7. Deeg, H.J. et al. (1986) Decreased incidence of marrow graft rejection in patients with severe aplastic anemia: changing impact of risk factors. *Blood* **68(6),** 1363–1368.
8. Deeg, H.J. et al. (1979) Abrogation of resistance to and enhancement of DLA-nonidentical unrelated marrow grafts in lethally irradiated dogs by thoracic duct lymphocytes. *Blood* **53(4)**, 552–557.
9. Storb, R. et al. (1982) Marrow transplantation with or without donor buffy coat cells for 65 transfused aplastic anemia patients. *Blood* **59(2)**, 236–246.
10. Kernan, N.A. et al. (1993) Analysis of 462 transplantations from unrelated donors facilitated by the National Marrow Donor Program. *N. Engl. J. Med.* **328(9)**, 593–602.
11. Hale, G., Cobbold, S., and Waldmann, H. (1988) T cell depletion with CAMPATH-1 in allogeneic bone marrow transplantation. *Transplantation* **45(4)**, 753–759.
12. Mitsuyasu, R.T. et al. (1986) Treatment of donor bone marrow with monoclonal anti-T-cell antibody and complement for the prevention of graft-versus-host disease. A prospective, randomized, double-blind trial. *Ann. Intern. Med*. **105(1)**, 20–26.
13. Antin, J.H. et al. (1991) Selective depletion of bone marrow T lymphocytes with anti-CD5 monoclonal antibodies: effective prophylaxis for graft-versus-host disease in patients with hematologic malignancies. *Blood* **78(8**), 2139–2149.
14. Prentice, H.G. et al. (1984) Depletion of T lymphocytes in donor marrow prevents significant graft-versus-host disease in matched allogeneic leukaemic marrow transplant recipients. *Lancet* **1(8375)**, 472–476.
15. Laughlin, M.J. et al. (2004) Outcomes after transplantation of cord blood or bone marrow from unrelated donors in adults with leukemia. *N. Engl. J. Med.* **351(22)**, 2265–2275.
16. Rocha, V. et al. (2004) Transplants of umbilical-cord blood or bone marrow from unrelated donors in adults with acute leukemia. *N. Engl. J. Med*. **351(22)**, 2276–2285.
17. Wagner, J.E. et al. (2002) Transplantation of unrelated donor umbilical cord blood in 102 patients with malignant and nonmalignant diseases: influence of CD34 cell dose and HLA disparity on treatment-related mortality and survival. *Blood* **100(5)**, 1611–1618.
18. Chao, N.J., Emerson, S.G., and Weinberg, K.I. (2004) Stem cell transplantation (cord blood transplants). *Hematology (Am. Soc. Hematol. Educ. Program)* 354–371.
19. Vormoor, J. et al. (1994) Immature human cord blood progenitors engraft and proliferate to high levels in severe combined immunodeficient mice. *Blood* **83(9)**, 2489–2497.
20. Broxmeyer, H.E. et al. (1992) Growth characteristics and expansion of human umbilical cord blood and estimation of its potential for transplantation in adults. *Proc. Natl. Acad. Sci. U. S. A.* **89(9)**, 4109–4113.
21. Broxmeyer, H.E. et al. (1989) Human umbilical cord blood as a potential source of transplantable hematopoietic stem/progenitor cells. *Proc. Natl. Acad. Sci. U. S. A.* **86(10)**, 3828–3832.
22. Szabolcs, P. et al. (2003) Coexistent naive phenotype and higher cycling rate of cord blood T cells as compared to adult peripheral blood. *Exp. Hematol*. **31(8)**, 708–714.
23. Barker, J.N. et al. (2005) Transplantation of 2 partially HLA-matched umbilical cord blood units to enhance engraftment in adults with hematologic malignancy. *Blood* **105(3)**, 1343–1347.
24. Kim, D.W. et al. (2004) Cotransplantation of third-party mesenchymal stromal cells can alleviate single-donor predominance and increase engraftment from double cord transplantation. *Blood* **103(5)**, 1941–1948.
25. Lin, M.T. et al. (2003) Relation of an interleukin-10 promoter polymorphism to graft-versus-host disease and survival after hematopoietic-cell transplantation. *N. Engl. J. Med.* **349(23)**, 2201–2210.
26. Takahashi, S. et al. (2004) Single-institute comparative analysis of unrelated bone marrow transplantation and cord blood transplantation for adult patients with hematologic malignancies. *Blood* **104(12),** 3813–3820.
27. Reid, C.D. et al. (1990) Identification of hematopoietic progenitors of macrophages and dendritic Langerhans cells (DL-CFU) in human bone marrow and peripheral blood. *Blood* **76(6)**, 1139–1149.
28. Korbling, M. and Anderlini, P. (2001) Peripheral blood stem cell versus bone marrow allotransplantation: does the source of hematopoietic stem cells matter? *Blood* **98(10),** 2900–2908.
29. Martin, P.J. (1993) Donor CD8 cells prevent allogeneic marrow graft rejection in mice: potential implications for marrow transplantation in humans. *J. Exp. Med.* **178(2)**, 703–712.
30. Panse, J.P. et al. (2003) Dog leukocyte antigen nonidentical unrelated canine marrow grafts: enhancement of engraftment by CD4 and CD8 T cells. *Transplantation* **76(3)**, 474–480.
31. Nimer, S.D. et al. (1994) Selective depletion of CD8+ cells for prevention of graft-versus-host disease after bone marrow transplantation. A randomized controlled trial. *Transplantation* **57(1)**, 82–87.
32. Chen, B.J. et al. (2004) Transfer of allogeneic CD62L- memory T cells without graft-versus-host disease. *Blood* **103(4)**, 1534–1541.
33. Anderson, B.E. et al. (2003) Memory CD4+ T cells do not induce graft-versus-host disease. *J. Clin. Invest*. **112(1)**, 101–108.
34. Zhang, Y. et al. (2004) Dendritic cell-activated CD44hiCD8+ T cells are defective in mediating acute graft-versus-host disease but retain graft-versus-leukemia activity. *Blood* **103(10),** 3970–3978.
35. Zhang, Y. et al. (2005) Alloreactive memory T cells are responsible for the persistence of graft-versus-host disease. *J. Immunol.* **174(5)**, 3051–3058.
36. Zhang, Y. et al. (2005) Host-reactive CD8(+) memory stem cells in graft-versus-host disease. *Nat. Med*. **11(12),** 1299–1305.
37. Bachar-Lustig, E., Reich-Zeliger, S., and Reisner, Y. (2003) Anti-third-party veto CTLs overcome rejection of hematopoietic allografts: synergism with rapamycin and BM cell dose. *Blood* **102(6)**, 1943–1950.
38. Steiner, D. et al. (2006) Tolerance induction by third-party "off-the-shelf" CD4(+)CD25(+) Treg cells. *Exp. Hematol.* **34(1),** 66–71.
39. Gandy, K.L. et al. (1999) CD8+TCR+ and CD8+TCR- cells in whole bone marrow facilitate the engraftment of hematopoietic stem cells across allogeneic barriers. *Immunity* **11(5)**, 579–590.
40. Fugier-Vivier, I.J. et al. (2005) Plasmacytoid precursor dendritic cells facilitate allogeneic hematopoietic stem cell engraftment. *J. Exp. Med*. **201(3),** 373–383.
41. Ildstad, S.T. et al. (2005) Preconditioning of NOD mice with anti-CD8 mAb and costimulatory blockade enhances chimerism and tolerance and prevents diabetes, while depletion of {alpha}{beta}-TCR+ and CD4+ cells negates the effect. *Blood* **105(6)**, 2577–2584.
42. Varadi, G., Friedman, T.M., and Korngold, R. (2005) A CD4 domain 1 CC' loop peptide analogue enhances engraftment in a murine model of bone marrow transplantation with sublethal conditioning. *Biol. Blood Marrow Transplant*. **11(12)**, 979–987.
43. Willemze, R. et al. (1992) In vivo use of Campath-1G to prevent graft-versus-host disease and graft rejection after bone marrow transplantation. *Bone Marrow Transplant.* **9(4)**, 255–261.
44. Hale, G. and Waldmann, H. (1994) Control of graft-versus-host disease and graft rejection by T cell depletion of donor and recipient with Campath-1 antibodies. Results of matched sibling transplants for malignant diseases. *Bone Marrow Transplant.* **13(5)**, 597–611.
45. Loren, A.W. et al. (2005) Intensive graft-versus-host disease prophylaxis is required after unrelated-donor nonmyeloablative stem cell transplantation. *Bone Marrow Transplant.* **35(9)**, 921–926.
46. Shlomchik, W.D. et al. (1999) Prevention of graft versus host disease by inactivation of host antigen-presenting cells. *Science* **285(5426),** 412–415.
47. Cao, T.M. et al. (2005) Engraftment and survival following reduced-intensity allogeneic peripheral blood hematopoietic cell transplantation is affected by CD8+ T-cell dose. *Blood* **105(6)**, 2300–2306.
48. Marks, D.I. et al. (2002) The toxicity and efficacy of donor lymphocyte infusions given after reduced-intensity conditioning allogeneic stem cell transplantation. *Blood* **100(9)**, 3108–3114.
49. Nathan, D.G. et al. (1978) Human erythroid burst-forming unit: T-cell requirement for proliferation in vitro. *J. Exp. Med*. **147(2)**, 324–339.
50. Monteiro, J.P. et al. (2005) Normal hematopoiesis is maintained by activated bone marrow CD4+ T cells. *Blood* **105(4)**, 1484–1491.
51. Goodman, J.W., Burch, K.T., and Basford, N.L. (1972) Graft-vs.-host activity of thymocytes: relationship to the role of thymocytes in hemopoiesis. *Blood* **39(6)**, 850–861.

## BIOSKETCH

**Elizabeth Hexner, M.D.** is an Instructor in the Department of Medicine, Division of Hematology/Oncology at the University of Pennsylvania in Philadelphia, PA. E-mail: elizabeth.hexner@uphs.upenn.edu
